# Facial Neuralgia

**Published:** 1904-09

**Authors:** 


### FACIAL NEURALGIA.

A NEW and simple method of relief for this condition (says *Health*) is brought forward by Dr. W. C. Belt. It is simply to direct the patient to place the hand, opposite the side on which the neuralgia is felt, in a basin of water as hot as can be borne. He claims that relief will be experienced in less than five minutes. His explanation of the action of this procedure is that the two nerves endowed with the greatest number of tactile nerve endings are the fifth and the median, and their motor areas in the cortex are not only adjacent, but actually overlap. As the fibres cross in the cord he expects a powerful tactile impulse conveyed from, say the left hand, to affect in some degree the cortical center of the fifth nerve of the opposite side.

The method is so simple that it may be tried in a number of cases, and if without benefit it will be without harm.—*Med. Times.*

